# Antipyrin in Puerperal Fever

**Published:** 1887-10

**Authors:** A. B. Anderson

**Affiliations:** Pawnee City, Nebraska


### ANTIPYRIN IN PUERPERAL FEVER.

BY A. B. ANDERSON, M. D., PAWNEE CITY, NEBRASKA.

Fever occurring during the puerperal state, whether due to septic infectious or malarial causes, is much to be dreaded, and any remedy or treatment that will greatly aid us in the successful management of these cases is highly appreciated by every practitioner.

Without going at all into the pathology or etiology of puerperal fever, it is our purpose to give some suggestions based upon experience in the treatment of fevers occurring in the puerperal state. Perhaps the most frequent form in this latitude is that of a malarial nature, but which is modified by the condition and surroundings of the puerpera until it becomes a much more serious case than one of a purely malarial nature uncomplicated by the puerperal condition. Again these cases may be increased in severity by a complication arising from septic influences. In all of these cases the temperature runs high, is difficult to control, and convalescence is tedious and subject to frequent relapses.

I claim for antipyrin especial praise in puerperal fever, not that it has any specific action, except that it is sure to control the high temperature, and in this direction alone is a very great adjunct in the treatment of this disease. In the first place it makes the patient much more comfortable by acting on all the secretions except the bowels. Under its use the kidneys respond promptly, the skin is bathed in perspiration, the secretions of the mouth are improved, the dry fissured tongue is moistened up, and a general feeling of comfort is induced. So uniform are these results that I confidently expect them to follow as certainly as purgation follows the administration of a dose of sulphate of magnesia. However, this does not cure the patient, by any means, and if this and other remedies were not continued, the same train of symptoms would reappear.

When there is septicæmia the patient may not recover, but I think her chances are increased ten-fold by the judicious administration of antipyrin. How often have we utterly failed in getting satisfactory results from the use of quinia in these cases! It may be because the secretions are locked up and the quinia is not absorbed and produces gastric irritation instead of sedation. The same is true of iron. The latter is useless, if not positively harmful, when there is high temperature, dry tongue and constipated bowels. Antipyrin acts like a charm in bringing about that condition of the system appropriate for the administration of quinia and iron.

In a case of puerperal fever which I had pronounced septic, in which there was great tenderness of peri-uterine and pelvic tissue, in which there was alarming dyspnœa. Night temperature (105) and chills, which failed to respond to 10 grains of quinia every six hours with alteratives and heart sedatives, made a good and every way satisfactory recovery, the initial step dating from the first dose of antipyrin. Below are my notes of this case, hurriedly kept:

Mrs. P., aged 26; married five years; first pregnancy; was confined on the 16th of January, 1887, attended by Dr. C. I was called on the 21st; patient had had a chill previous night; found her as follows: dorsal decubitus, great tenderness over lower part of abdomen, especially in right illiac region, temperature 104°, pulse weak and frequent, countenance pale and anxious, complaint of difficulty in breathing. Examination showed no trouble with the lungs. Diagnosis, puerperal fever.

*Treatment.*—Gave opium for the pain, quinia and digitalis for the pyrexia and hot fomentations for the bowels.

22d. Found the patient feeling somewhat better, pulse stronger, countenance brighter, temperature 103°.

23d. Patient worse, temperature 104°, pulse 130 and weak, difficult breathing, excessive tenderness over lower part of abdomen, no general peritonitis. Vaginal douches of hot carbolized water were ordered previous day, but not very well carried out. Lochia virtually stopped, vagina hot and dry. Gave to-day 5 grains quinia with 5 drops tinc. ferri chlorid. every four hours, with digitalis and whisky alternated.

January 24th, patient slightly better, temperature 103°, pulse 86, and feels more comfortable, unable to pass water; use the catheter and continue former treatment. Was called again in the night; attendants thought the patient dying. She had evidently had a chill, which was followed by high fever and oppressed breathing.

25th. Continued treatment.

26th. Same treatment continued.

27th. Patient so disgusted with quinia in the various forms in which I had administered it that she refuses to have any more of it; also refuses milk and whisky; changed to 10 grains of quinia dissolved in hydrochloric acid every six hours, giving some grape wine instead of the whisky.

28th. Had a prolonged chill from 5 to 8 p. m.; temperature 104° at 6 o'clock, and patient complaining of being cold; 10 grains of quinia were given at 6 p. m., dissolved in hydrochloric acid; vaginal discharge purulent and offensive; catheter used twice daily, and turpentine stupes to abdomen.

29th. Same condition and no particular change in the treatment.

30th. No chill to-day, but high temperature (105°), great depression, pulse 120, and weak.

31st. No change, temperature 104°, countenance anxious, watches every move I make, evidently trying to read my prognosis; temperature has not been under 104° at any time during the last three days. Patient has had 10 grains of quinia every six hours during that time. Somewhat cinchonized. Determined to drop the quinia, as it has utterly failed to control the high temperature. Gave 10 grains of antipyrin at 1 p. m., and waited its action. At 3 p. m. temperature was down to 102° and patient feeling better. I directed the nurse to repeat the dose at 6. p. m. if the temperature was not below 102°; left also sol. ferri nitrate to be given in 5-grain doses every three hours.

February 1st. Found the patient feeling much better, is stronger, and has taken some nourishment with relish; temperature 102°; nurse says temperature was down below 100° all night and rested well. I ordered 5 grains antipyrin and repeated at intervals of four or six hours to keep the temperature at or below 100°.

2d. Patient still better, temperature 100½° at 4 p. m., pulse 96, skin moist, has relish for food, takes buttermilk and gruel; has taken three 5-grain doses of antipyrin in the last twelve hours; has had no quinia in forty-eight hours, and has had no chill. Kidneys act naturally to-day; some pain; bowels move well every other day by enema.

3d. Patient doing well every way.

Was called in the night to use catheter. Drew half pint of rather highly colored urine. Prescribed nitre, tr. iron, and nux vomica; temperature, 98½°, pulse, 96. From this time the patient made a rapid and uninterrupted recovery.

This, with a number of other cases, has proven to my mind that in antipyrin we have an invaluable remedy in puerperal fever. Say what you may about removing the cause of disease, experience plainly teaches us that we must attack the symptoms. In a great many of these cases the fever stands as a barrier before us in our attacks upon the disease. And when we control this condition, we save the powers of the patient to resist and meet other complications that may arise. When Dr. Thomas would use the rubber coil, I would use antipyrin. Dr. Fordyce Barker recommends when quinia fails to control the pyrexia, to use Warburg's Tincture. I would say, when quinia fails, and it generally will fail in puerperal cases, give antipyrin. If you give it as a specific you will be disappointed, but if you give in moderate doses to bring down the fever, to moisten the skin, to act on the kidneys, to increase the secretions of the mouth, you will not be disappointed, or your experience will not accord with mine.—*Peoria Medical Monthly.*

